# Gene Therapy in Crohn’s Disease: Current Preclinical Challenges and Future Translational Avenues

**DOI:** 10.3390/biomedicines13123078

**Published:** 2025-12-13

**Authors:** Solafah Abdalla, Antoine Brouquet, Romina Aron-Badin, Pierre Bougnères

**Affiliations:** 1 Service de Chirurgie Viscérale et Oncologique, Hôpital de Bicêtre, Assistance Publique-Hôpitaux de Paris, 94270 Paris, France; antoine.brouquet@aphp.fr; 2 Service de Chirurgie Viscérale et Oncologique, Faculté de Médecine, Université Paris-Saclay, Le Kremlin-Bicêtre, 94270 Paris, France; 3 Laboratoire des Maladies Neurodégénératives, MIRCen Institute, Commissariat à l’Energie Atomique, Fontenay-aux-Roses, 92260 Paris, France; romina.aron-badin@cea.fr; 4 Therapy Design Consulting, Vincennes, 94300 Paris, France

**Keywords:** gene therapy, Crohn’s disease, enteric nervous system

## Abstract

Crohn’s disease (CD) remains a highly complex disorder, and the progress of preclinical gene therapy for CD has been constrained by several significant challenges. These include the identification of optimal therapeutic gene targets, the difficulty of targeting therapy-resistant cells within a chronic inflammatory microenvironment, particularly in the enteric nervous system (ENS), and the lack of robust animal models that faithfully recapitulate human pathology, as classical models largely rely on toxin-induced colitis. This review synthesizes major preclinical studies on gene therapy for CD and related inflammatory bowel diseases (IBD). We critically assess the rationale and biodistribution data for different vector platforms, considering vector type, promoter, and route of administration, in the ileum and colon of both rodent and non-human primate models. Special attention is given to strategies targeting the ENS. Finally, we explore the putative therapeutic aims of these approaches, including direct attenuation of intestinal inflammation and prevention of postoperative recurrence of CD via local intraoperative gene delivery. Although most data derive from chemical colitis models, this review establishes a foundational framework to inform translational research in gene therapy for CD and other IBDs.

## 1. Introduction

Inflammatory bowel diseases (IBD), particularly Crohn’s disease (CD), are chronic, debilitating conditions caused by intestinal and systemic inflammation. Despite significant advancements in conventional treatments, including immunosuppressants and monoclonal antibodies [1], approximately 50% of CD patients still require bowel resection within ten years of diagnosis due to intestinal strictures or penetrating complications [2]. The most frequent surgical procedure is ileocolic resection. Unfortunately, postoperative recurrence is common, with half of patients developing recurrent symptoms and about one-third requiring repeat surgery within five years of the initial resection [2]. Postoperative recurrence is often clinically silent; indeed, although 70% to 90% of patients show endoscopic disease recurrence within one year after surgery, only a minority report symptoms [3].

Gene therapy, a promising strategy for many monogenic disorders, faces unique challenges in CD due to its polygenic and multifactorial pathology. This complicates the selection of a suitable therapeutic gene capable of effectively modulating intricate inflammatory pathways. Preclinical gene therapy development in CD is hindered by three major factors: (i) absence of representative models: classical animal models rely on chemically induced colitis and fail to fully replicate CD’s multifaceted human pathology; (ii) cellular targets of gene therapy are yet to be validated: indeed, therapeutic vectors must ensure sustained transgene expression in cells resistant to chronic inflammation; in this respect, the enteric nervous system (ENS), emerges as a key target; (iii) the justification that gene therapy can provide significant benefits beyond effective existing therapies.

Despite these hurdles, the potential for local, durable therapeutic gene expression within the inflamed gut, particularly targeting the ENS, provides a compelling rationale for the continued development of gene therapy attempts.

This review systematically examines early preclinical gene therapy studies for CD and analyzes the rationale behind vector platform selection, including choice of therapeutic gene, promoter, and administration route. It also evaluates biodistribution of vector expression in ileal and colonic tissues of rodents and non-human primates. The review also explores two different clinical scenarios having therapeutic promise: direct anti-inflammatory treatment during active disease or prevention of postoperative recurrence via vector injection into preserved intestinal segments near the ileocolic anastomosis.

By synthesizing current preclinical data, this review aims to establish a focused framework able to guide translational research in gene therapy for IBD (CD and Ulcerative colitis), highlighting both the challenges and transformative potential of this field.

## 2. Genes Predisposing to CD

From a gene therapy perspective, it is useful to recall the current knowledge regarding the genetic determinants of the disease. To date, no form of CD, nor even a “pure” Crohn-like phenotype, has been identified as being caused by the loss of function of a single gene [4,5,6]. The genetic causality of CD involves more than 125 germline variants identified through genome-wide association studies (GWAS) that have been recently reviewed [7,8,9,10]. These variants, which are widespread in the general population, are not amenable—by virtue of their number and their predominantly non-coding nature—to the replacement by a normal coding sequence, as achieved by gene therapy. They have no established function and are thus associated with CD, not directly causative. They are thus not prone to gene editing approaches. Among the associated variants, nine emerged as highly related to diseases and biological functions including NOD2, TNF, ICAM1, NFKBIA, NFKB1, TNFRSF1A, CD14, ACE TLR4, and TLR9 which are involved in both TNF and of NF-κβ signaling [10]. Also, IL10, IFNG, IL1B, IL6, IL23R, IL4, IL12B, IL1RN, IL18, and IL4R, which contribute to the inflammatory response and interaction of cytokines [10]. None of these variants, however, exerts a predominant effect, the strongest among them conferring only a relative risk of less than 1.5 [8,10]. Epigenetic marks regulating the expression of several of these genes have been identified in circulating cells as well as within the inflamed intestinal region [11,12].

In addition, somatic mutations identified within colonic crypts [13] further contribute to the genetic heterogeneity of CD. Given this considerable complexity, it is unsurprising that no compelling gene therapy strategy based on the replacement of a single therapeutic gene has yet emerged. Nevertheless, gene therapy approaches designed to deliver genes capable of counteracting deleterious mechanisms within immune and cytokine networks remain promising. The goal of such approaches would be to attenuate the intestinal inflammation that leads the majority of patients to require ileocolic resection.

In patients requiring ileocolic resection, another relevant potential application of gene therapy in CD would be the prevention of postoperative recurrence of inflammation. From a gene therapy standpoint, it is noteworthy that postoperative recurrence has been associated with low local concentrations of IL-10 [14]. This phenomenon has also been explored through genetic investigations [15]. More informative than peripheral blood gene sequencing, gene expression studies conducted on ileal mucosal samples, either from the proximal margin of resected specimens or collected during postoperative endoscopy, have demonstrated that early endoscopic recurrence is typically accompanied by upregulation of the TNFα, IFNγ, IL23A, and IL17A genes [16]. However, the predictive value of these overexpressed genes for recurrence risk remains limited and is complicated by marked inter-individual variability. A plausible interpretation is that these molecular alterations are more likely consequences, rather than causes, of the local inflammation that necessitated surgical intervention.

## 3. The Main Features and Players on the Inflammatory Battlefield

Depending on the clinical course and the degree of inflammation, biopsies from various intestinal segments are used to classify CD as “active,” “chronic active,” or “chronic inactive.” Active disease is characterized by dense infiltration of the lamina propria, surface epithelium, and crypt lumina (cryptitis). The inflammatory infiltrate consists predominantly of lymphocytes, plasma cells, and macrophages, accompanied by granulocyte invasion of the lamina propria and crypts, leading to crypt abscesses, hemorrhage, erosions, ulcerations, and necrosis [17,18,19]. Histological features of chronicity include distortion of the mucosal architecture with irregular and atrophic crypts [20], diffuse lamina propria infiltration with basal plasmacytosis and lymphoid aggregates, and epithelial changes such as mucin depletion, surface injury, and Paneth cell metaplasia. Transmural extension of lymphoid aggregates [18,19] and inflammatory cells into the submucosa and muscularis may give rise to deep ulcers, and fibrosis. During remission, the mucosa may either regain a normal appearance or remain atrophic [21]. A characteristic hallmark of CD is the coexistence of chronic inflammation and focal crypt distortion adjacent to normal crypts [20].

Beyond the above description of inflammatory lesions, the question arises as to the mechanisms that cause them. Over the last decade, a major player in the pathology proved to be the ENS, commonly referred to as the “second brain”. Intercalated between the mucosal and muscular layers throughout the length of the gastrointestinal tract, ENS consists of hundreds of millions of enteric neurons and enteric glia organized into two ganglionated plexuses called the submucosal (SMP) and myenteric plexus (MP), respectively. The MP is located between the circular and longitudinal muscle and the SMP just below the mucosa in the lumen of the gut. Their neurons are connected to the central nervous system (CNS) and also belong to integrative complex circuits independent of CNS connections. Other component of ENS are the enteric glial cells (EGCs) [22,23,24]. EGCs represent a complex and fascinating population of cells distributed across the different layers of the intestinal wall. For a detailed overview of current knowledge on EGCs, two excellent review articles can be consulted [22,25].

**Figure 1:**
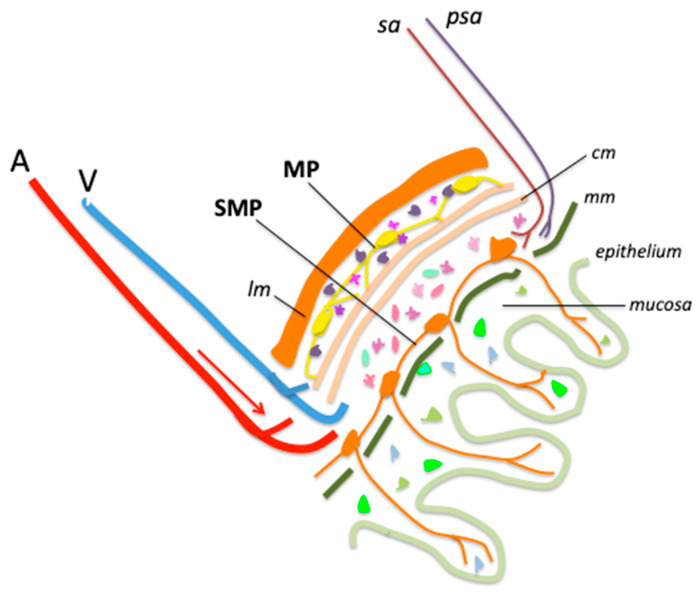
Main structures and cells of healthy ileum and colon wall. A, artery; V, vein; lm, longitudinal muscle; SMP, submucosal plexus; MP, myenteric plexus; sa, sympathetic axon; psa, parasympathetic axon; cm, circular muscle; mm, muscularis mucosa. Enteric glial cells (EGCs) are figured as small multicolored cells distributed across all layers of the intestinal wall.

We have illustrated, in a simplified diagram, the main structures of the abdominal wall, highlighting the position of the MP and SMP (Figure 1).

The nature and functions of EGCs are only beginning to be understood. Comparisons have been drawn with glial cells of the CNS and peripheral nervous system (PNS), which have been far more extensively studied, but the similarities between these cell populations remain limited. Unlike CNS or PNS glia, EGCs do not produce myelin [26,27,28], although they are capable of synthesizing myelin-associated proteins [29] such as PLP1 [30]. Commonly used markers to identify EGCs include the glial fibrillary protein (GFAP), the calcium binding protein S100b, proteolipid protein 1 (PLP-1), and the transcription factor SRY-Box Transcription Factor 10 (SOX10) [30]. GFAP-positive EGCs have been compared to astrocytes [28] but there is no evidence that they share similar functions [22]. Indeed, it is not known whether EGCs have a major role in astrocytic functions such as ion homeostasis, pH regulation, chemosensing, regulation of energy balance, control of blood–brain barrier, regulation of local blood flow, or glycogen storage [22,25].

EGCs are a heterogeneous and plastic population, whose gene expression profile can adapt to physiological or pathological changes in the local environment. Comparative analyses of human and mouse glial gene expression signatures demonstrate a high degree of consistency between human and murine EGCs [22,25]. At any given time, EGCs may adopt configurations that extend well beyond the conventional classification based solely on their morphology and anatomical localization within the intestinal wall [22,31,32,33]. This scheme distinguishes EGCs that encircle neuronal cell bodies with short processes within the MP and SMP, those that extend long processes connecting myenteric ganglia, those simultaneously located in the mucosa and in the MP or SMP, and those embedded within the smooth muscle layers. However, such a classification does not fully encompass the remarkable functional diversity of EGCs across different intestinal regions [34].

No single glial marker can yet identify a particular EGC subtype, given regional variation, time dependence and cell-type specificity in marker expression. For example, the expression of GFAP is dynamic and varies depending on the glial state [35] and subtype [31]. About 20% to 40% of glial cells that are extra-ganglionic express only GFAP [30]. GFAP has been used as a marker of EGCs in only one AAV biodistribution study [36].

The functions of EGCs, which remain only partially understood, are diverse and mediated through their interactions with other cell types of the intestinal wall. EGCs not only secrete key signaling molecules but also receive cues from neighboring cells. In relation to neurons, EGCs provide support to myenteric and submucosal neurons and regulate the activity of enteric neural circuits [22,25]. They have been shown to act as neural stem cells of the gut, as a subset of EGCs can differentiate into enteric neurons in response to ENS injury [37,38,39]. EGCs also sustain neurotransmission by modulating neurotransmitter availability, controlling oxidative stress, providing neurotrophic factors, and promoting neurogenesis [22,25].

With respect to enterocytes, EGCs influence the epithelial stem cell niche, where glial-derived factors regulate epithelial maturation and differentiation [40,41,42]. Mucosal EGCs communicate with enterocytes through glial cell line-derived neurotrophic factor (GDNF) secretion and interact with enteroendocrine cells. Notably, GDNF expression is upregulated in CD biopsies [25]. In addition, EGCs release epidermal growth factor (EGF) and transforming growth factor-β (TGF-β) in response to inflammatory stimuli [43,44].

EGCs are also directly involved in the intestinal inflammatory response. Upon exposure to inflammatory signals, specific EGCs subtypes rapidly enter a reactive state called “gliosis,” characterized by changes in molecular profile, structure, and function [35]. Increased expression of GFAP and S100β has been observed in inflamed colonic biopsies from CD patients [45]. In chronic inflammation, EGCs interact with innate lymphoid cells (ILCs), which are regulated by numerous inflammatory mediators, such as neuropeptides, hormones, eicosanoids, and cytokines [46,47,48,49,50]. EGCs also play a crucial immunoregulatory role in inflammatory conditions through their interaction with the adaptive immune systems [51,52]. EGCs exchange signals with macrophages in response to inflammation, a key mechanism in chronic colitis [49]. Pro-inflammatory signals such as IL-1b, induce glial reactivity [25]. The complex interplay of glia, innate lymphoid cells, and epithelial cells modulate intestinal homeostasis, inflammation, and defense [53]. EGCs contribute to the maintenance of the intestinal epithelial stem cell niche, a crucial function for the repair of the intestinal epithelium upon colitis. EGCs display dysfunctional responses in patients with CD [54,55,56] and in the mouse model of dextran sodium sulfate (DSS) colitis [49,57,58,59]. During DSS-induced colitis, EGCs and neurons are exposed to inflammatory processes and molecules, once intra-ganglionic macrophages induce degradation of the membrane surrounding the MP [59]. In CD, myenteric plexi and the periganglionic areas are infiltrated by T and B lymphocytes and granulocytes («plexitis») [60,61]. These immune cell infiltrations are present in the mouse model of chronic DSS-induced colitis [60]. Interestingly, studies of resection specimens have consistently shown that plexitis contributes to the prediction of post-operative recurrence of the disease [62,63,64].

EGCs can serve as a relay between psychological stress and gastrointestinal inflammation [65]. During periods of stress, an inflammatory subset of EGCs is generated, called enteric glia associated with psychological stress (eGAPS). These eGAPS then produce colony stimulating factor 1 (CSF1), which triggers the production of TNF by monocytes. This TNF production exacerbates intestinal inflammation [65].

Since CD patients are at increased risk of developing colorectal cancer (CRC) [66], the potential contribution of EGCs to this predisposition has been questioned [67,68,69]. EGCs may influence both the development and progression of CRC [25], acting as key components of the tumor microenvironment. Indeed, they have been shown to promote tumor growth. Three-dimensional imaging of colonic adenocarcinomas revealed that S100β^+^ GFAP^+^ enteric glial cell bodies and processes extensively infiltrate the tumor mass [70], including direct interactions between EGCs and tumor-associated macrophages within the CRC microenvironment [71].

In conclusion, despite significant advances, several challenges remain in understanding how EGCs function and delineating their contributions to disease pathogenesis. Molecular mechanisms underlying EGCs origin, heterogeneity, plasticity, specialization, immune modulation and tissue damage or repair demands further exploration. Understanding the precise contributions of EGCs to complex disorders like CD and CRC might offer an opportunity to identify potential therapeutic targets [22,25].

## 4. Current Treatments for CD

TNF inhibitors (anti-TNF) represent the preferred first-line advanced therapy for moderate-to-severe CD [1]. Nevertheless, an inadequate response to a given anti-TNF or the occurrence of unacceptable adverse effects frequently necessitates either switching to another anti-TNF or initiating an advanced therapy with a different mechanism of action. Over the past decade, substantial progress has been achieved in the medical management of CD [72,73,74,75,76,77]. However, the efficacy of these therapies remains limited: while anti-TNF agents achieve an initial clinical response in approximately 70 to 80% of patients, as demonstrated in pivotal trials such as SONIC [78] and early combined immunosuppression strategies [79], only about 40% of patients achieve and sustain clinical remission in real-world settings [80,81]. Furthermore, long-term treatment durability is hampered by a progressive loss of efficacy, estimated at nearly 10% per patient-year [81,82]. The majority of CD patients ultimately require intestinal resection within 10 years of diagnosis, with an even higher likelihood among those with ileal involvement [7,83,84,85,86]. Indeed, despite the increasing number of patients receiving biologics, the rates of ileocolic resection and the extensiveness of resections has remained stable over time [87]. Cumulative risk of re-resection after initial surgery is nearly 10% at 5 years, 20% at 10 years and 30% at 20 years [88].

Several studies have notably shown that anti-TNFα does not reduce clinical post-operative recurrence [89]. The POCER study suggested that adalimumab could be superior to thiopurines in preventing early endoscopic recurrence in high-risk patients [90]. Newer therapies, such as ustekinumab and vedolizumab, can rescue patients with medically refractory CD, but their preventive effect on post-operative recurrence is yet untested [91].

## 5. Challenges and Prospectives for the Gene Therapy of CD

This section will primarily address gene therapy approaches based on viral vectors. Gene therapy requires the design of a vector consisting of a cDNA cassette packaged within a viral capsid, which enables delivery to the nucleus of target cells, where the selected promoter drives expression of the therapeutic gene at a sufficient level. The success of this strategy depends on both the capsid tropism and the local concentration of the vector, which in turn is determined by the route of administration. For potential application to human disease, additional critical requirements include sustained expression of the therapeutic gene, as well as patient tolerance of both the viral vector load and the transgene. A limited number of studies have paved the way for gene therapy in CD.

### 5.1. Selection of a Therapeutic Gene

There is yet no definite answer to this dilemma (see Section 2). A rational choice would be to select a gene able to inhibit the main inflammatory mechanisms of CD and to prevent postoperative recurrence. Genes coding for cytokines such as IL-10, TGFβ, IL-37 and IL-38 are potential candidates. Among them, IL-10 may appear as a prominent candidate. It is considered the most important anti-inflammatory cytokine in humans, produced by macrophages, dendritic cells, B cells, and other cells. The importance of IL-10 in the regulation of the intestinal immune system is demonstrated in IL-10 deficient and IL-10 receptor-2 deficient mice by the development of a T helper-1-mediated intestinal inflammation [92,93]. The counter-regulatory role of IL-10 towards mucosal inflammation is multifactorial. IL-10 is a potent down regulator of IL-12 production, thus acts on Th1 cell induction [94]. In addition, IL-10 suppresses the production of other pro-inflammatory cytokines including TNFα IL-1β, IL-6 and IL-8 [95]. Finally, there is substantial evidence that IL-10 acts to promote the differentiation and the activity of regulatory T cells [96]. The observations in IL-10^−/−^ mice led to therapeutic trials of IL-10 in other models of colitis, showing that systemic IL-10 administration can prevent intestinal inflammation by down regulating the intestinal pro-inflammatory Th1 response [92,97,98]. Based on these successful experimental findings, recombinant (r)IL-10 was administered by subcutaneous injection to patients with either mild/moderate or steroid refractory CD, or in patients undergoing ileal resection to prevent postoperative recurrence [99,100,101]. Systemic rIL-10 therapy appeared safe and well tolerated, but resulted in only a modest response. Explanations for this lack of efficacy include the short half-life of rIL-10 [102], local delivery of insufficient amounts of rIL-10 to inhibit mucosal Th1 responses and the side effects associated with high-dose rIL-10 [103]. It is noteworthy that inflammatory involvement of the gastrointestinal tract manifests early in life in cases of IL-10 deficiency, both in humans and in murine models [8,9,10]. Similar early inflammatory phenomena have also been described in other disorders affecting immune or inflammatory responses.

Other anti-inflammatory cytokines or regulatory pathways under investigation might also provide relevant therapeutic transgenes. Manipulations of other cytokines were attempted in colitis mouse models. Adenovirus (AdV)-induced overexpression of oncostatin M (OSM), a multifunctional cytokine that belongs to the IL-6 subfamily, improved clinical scores in a DSS colitis mouse model [104]. This finding in a rodent model contrasts with the increased concentration of OSM found in inflammatory bowel diseases, suggesting a deleterious action of this cytokine [105]. Another study showed the beneficial effects of AdV-mediated IL-4 overexpression in a rat TNBS colitis model [106] whereas IL-4 gene transfer to the healthy intestine triggers inflammation [106,107]. Silencing a pro-inflammatory cytokine can also be beneficial. For example, silencing IL-18 using AdV-delivered antisense RNA reduced inflammation in a T cell transfer colitis model [108]. Although studies of cytokine overexpression or silencing with viral vectors provide a variety of treatment options, the results obtained in rodent colitis models may not apply to patients with CD. Indeed, the disruption of the delicate cytokine balance may give contrasted results. For example the systemic expression of IL-10 induced by AAV-based gene therapy in rodent models carries its own risks of splenomegaly and thrombocytopenia [109]. An efficient and safe IL-10 based gene therapy of CD would need a delicate balance between intestinal production of IL-10 and a limited increase in circulating IL-10 concentrations. For this purpose, choice of capsid and promoter should both tune intestinal IL-10 expression to a favorable level, and restrict extra-intestinal IL-10 production by extra-intestinal tissues, notably the liver.

### 5.2. Selection of a Promoter

Beyond vector penetration and intracellular trafficking, promoters drive transgene expression in target cells. There are two categories of promoters. Promoters considered «ubiquitous» are supposed to control transgene expression in most if not all body cells. The other category comprises cell-specific or cell-restricted promoters. The usage of ubiquitous and tissue-specific promoters varies across therapeutic areas. Selection of a promoter thus obeys a functional objective based on the transgene action on the biology of cells implicated in the disease pathophysiology. Promoter selection should also cope with the packaging capacity of the capsid given the size of the transgene. Indeed AAV vectors cannot package more than 5 kb of cDNA encompassing the transgene and the promoter. While capsid engineering is being extensively explored to target specific cell types, efforts to find promoters have been less extensive. One reason is that well-established universal promoters are able to drive strong gene expression in various mammalian host cells. Indeed, a handful of promoters, the chicken β-actin (CBA) (around 1.6 kb) (CB refers to hybrid or shortened versions), and shorter cytomegalovirus (CMV), CBh (CBA hybrid), short CMV early enhancer/chicken β-actin/short β-globin intron (sCAG) and its shortened versions, mouse phosphoglycerate kinase (PGK) and human synapsin (SYN) promoters are commonly used for driving transgene expression in the nervous system [110]. The CBh and CBA promoters exhibit key differences in cell-specific activity in the nervous system of rodents. Indeed, the CBA promoter tends to drive highly neuron-specific expression, whereas the CBh promoter supports more balanced expression between neurons and oligodendrocytes. The CBh promoter generally provides strong, long-term, and ubiquitous CNS expression, often at higher levels than the CBA promoter. CMV early enhancer/chicken β-actin/short β-globin (CAG) drives high-level expression in most cell types. Recent surveys showed that over 50% of rAAVs in 106 clinical settings used the CMV, CBA and CAG promoters, considered to be ubiquitous [111,112]. These promoters have strong ubiquitous activity in various cell types, their size ranges from 500 (CMV) to 1000 bp (CAG) and larger, and such large sizes can be disadvantageous for rAAV gene therapy because the maximum packaging capacity of rAAVs in the range of 5.2–5.6 kb. Much smaller promoters could deliver larger cargo cDNAs as well as improve the packaging efficiency and vector titers [113]. The packaging capacity of AAVs is much smaller than other viral vectors such as lentivirus (LV) or AdV.

An alternative category of promoters are the cell-specific promoters, cited here for glia transduction [110]. Astrocyte-specific promoter studies have mostly focused on the GFAP promoter (2.2 kb) and its shortened versions. Additional candidates for astrocyte-specific promoters have also been suggested [114], including *Slc1a3*, which codes for the glutamate transporter SLC1A3, or *Gjb6*, which codes for the gap junction protein Connexin30 only expressed in gray matter astrocytes. Oligodendrocyte-specific promoters have also been developed. The large size and poor oligodendrocyte specificity of the myelin basic protein (*Mbp*) promoter [115] limits its usefulness. The myelin-associated glycoprotein (MAG) drives AAV9-carried transgene expression not only in oligodendrocytes [115,116,117] but, unexpectedly, in white matter astrocytes of mouse spinal cord and cerebellum [116,117]. A CBh promoter can shift gene expression from striatal neurons to oligodendrocytes [118].

Because oligodendrocyte promoters share transcription factor binding motifs, they can also activate transgene expression in Schwann cells. Their large size, however, generally prevents their incorporation into AAV constructs. For instance, while the full-length *Mbp* promoter supports expression in Schwann cells, its truncated 1.3–1.9 kb fragments, sufficient for oligodendrocyte targeting, lack the enhancer sequences necessary for expression in Schwann cells [119]. Similarly, the 2′,3′-cyclic-nucleotide 3′-phosphodiesterase (*Cnp*) and proteolipid protein (*Plp*) promoters have been shown to be expressed in Schwann cells, and a *Cnp* promoter has been used to drive expression in oligodendrocytes using LV, but are too big to use with AAV [120]. The 0.3 kb fragment of the *MAG* promoter is able to drive expression in Schwann cells in adult mice and non-human primates [116,117]. The myelin-specific myelin protein zero (*Mpz*) promoter (1.1 kb) shows strong Schwann cell selectivity. It has been successfully applied with LV vectors [121] and represents a promising candidate for future AAV-based strategies.

Satellite glial cells, located in peripheral sensory, sympathetic, and parasympathetic ganglia, are closely associated with neurons and appear to fulfill roles analogous to those of astrocytes in the CNS. Transgene expression in these cells can be achieved with high specificity using a GFAP promoter [122].

Although enteric neurons can be efficiently targeted using promoters such as CMV, CBA, or CBh, no promoter with specificity for enteric glia has yet been identified. Transcriptomic analyses indicate that EGCs are developmentally more related to oligodendrocytes and Schwann cells than to astrocytes [123]. Within the intestinal environment, however, they acquire an astrocyte-like phenotype, evidenced by GFAP expression, which is absent in Schwann cells [124]. This phenotypic divergence likely contributes to the relatively poor transduction efficiency of enteric glia compared with central nervous system glia. The development of an enteric glia-specific promoter therefore remains a critical unmet need.

### 5.3. Selection of a Vector

This section will not present viral vectors in the historical order in which they have been tested in the intestine of animal models, mostly rodents, over the past 25 years. It will instead start with AAV vectors, which currently represent the most widely used platform for gene therapy programs.

#### 5.3.1. Adeno-Associated Virus (AAV)

Adeno-associated virus (AAV) are small, non-enveloped, single-stranded DNA viruses belonging to the Parvoviridae family. They can only package expression cassettes no larger than 5 kb, AAV is currently the safest and most effective gene delivery vector owing to its low immunogenicity, low insertion rate, ability to transduce a broad range of host cell types, and capability of long-term transgene expression. Gene therapies based on recombinant AAV vectors have gained marketing approval both in the European Union and in the United Sates to treat a few human disorders, such as hemophilia A and B, inherited blindness, and spinal muscular atrophy, among others. The knowledge acquired using AAV in over 200 clinical trials demonstrates its remarkable safety and efficacy profiles [125,126,127] and confirmed its high potential as a platform to deliver gene therapeutics.

#### 5.3.2. Adenoviral (AdV) Vectors

Adenoviruses are non-enveloped and non-integrating viruses that can infect both dividing and non-dividing cells. The third generation of AdV vector leaves a large space for transgene packaging. AdV can infect intestinal epithelial cells with high efficiency [128]. After IV administration, AdV target the liver and the spleen, but also the colon [129,130]. In two landmark studies [131,132], Lindsay et al. demonstrated that the systemic administration of AdV vectors expressing mouse IL-10 reduces clinical and histological signs of 2,4,6-trinitrobenzene sulphonic acid (TNBS) colitis, the murine model for inflammatory bowel diseases known to be poorly responsive to systemic injections of recombinant IL-10. Two decades ago, these mouse data suggested that AdV-mediated gene therapy could be beneficial to patients with CD.

However, AdV vectors induce strong immune reactions which can result in serious adverse effects and limit the duration of transgene expression. Also, most people have already been exposed to AdV and possess neutralizing antibodies. Another limit is the short gene expression of these non-integrative vectors. Last but not least, the toxicity of AdV vectors raised significant concerns. While efforts have been made to engineer improved adenoviral vectors with reduced immunogenicity or alternative serotypes [133,134,135,136,137,138,139,140,141], their clinical applicability remains restricted by lingering safety concerns. In summary, immunogenicity, pre-existing immunity, transient effects, and safety concerns have caused a shift away from AdV vectors for gene therapy in favor of other viral vector platforms.

#### 5.3.3. Retroviral Vectors

Gamma retroviruses are enveloped RNA viruses that can introduce their genome into target cells and be retrotranscribed to double-stranded DNA prior to integration into the host genome. Therefore, these viruses guarantee sustained expression of the transgene. Their large genome size allows the efficient transfer of up to 10 kb of genetic material to host cells. Initially, recombinant retroviral vectors used either natural or synthetic viral elements to drive high transgene expression. However, soon after transplantation of the modified cells, it appeared that the integration capacity might result in insertional mutagenesis and uncontrolled proliferation.

The first reports of in vivo gene transfer into the intestine by retroviral vectors emerged in the 1990s with the Moloney murine leukemia virus (MLV) being introduced in the ileum lumen of rats and mice. Application of retroviruses to intestinal diseases has been reviewed by Buckinx et al. [142]. Reporter gene expression in the intestinal epithelium was described 4 days after retroviral vector administration [143] in accordance with studies showing that intestinal epithelial cell lines can be transduced by retroviral vectors [144,145,146,147,148]. However, the in vivo or ex vivo transduction efficiency was very low for MLV [143,146,149,150]. In vitro experiments in Caco-2 cells showed that a vesicular stomatitis virus G protein-pseudotyped (VSV-G) lentiviral approach was more efficient than VSV-G-MLV [147]. Self-inactivating (SIN) retroviral vectors have now been largely replaced in clinics by the more effective LV vectors with the same SIN configuration. LV belong to the genus of retroviruses and possess two copies of a single-stranded RNA genome. The basic structure of LV is based on the human immunodeficiency virus type 1. LV can infect both dividing and non-dividing cells as its genome can cross the nuclear membrane. This feature together with limited immunogenicity, has contributed to the widespread use of LV for a variety of pre-clinical and clinical applications. Even though LV have a slightly reduced preference to integrate in proximity to gene transcription start sites as compared with gamma-retroviruses, they share a similar integration mechanism and therefore entail the same concerns of insertional mutagenesis [151]. The adoption of the SIN strategy, as seen for retroviral vectors, has significantly improved LV safety [152,153,154]. Notably, integrase-defective lentiviral vectors (IDLV) have been developed for reducing viral genome integration by introducing specific mutations in the viral integrase gene [155].

#### 5.3.4. Non-Viral Strategies

Non-viral strategies can transfer genetic cargo to cells and organisms [151,156] based on molecules capable of forming complexes with the cargo with the double role of protecting it and shuttling it to the cytosol of the target cell via endocytosis. The expression of a transgene delivered by non-viral vectors is generally transient. This is because most of these vectors (naked DNA, plasmids, liposomes, polyplexes, etc.) do not integrate the genetic material into the host cell genome. As the therapeutic transgene remains in an episomal form, it is progressively degraded or diluted during cell divisions, leading to a rapid decline in transgene expression. Expression may persist for a few days to several weeks in renewing tissues, and occasionally longer in post-mitotic tissues such as certain neurons. However, long-term stable expression is generally not achieved.

### 5.4. Vector-Targeted Cells

The selection of target cells is a complex determinant that governs the choice of vector type, promoter, and ROA, each of which depends on the specific function the transgene is designed to alter.

Epithelial cells of the intestinal mucosa have traditionally been the primary targets, particularly through the use of AdV. Numerous studies have demonstrated successful transduction of epithelial cell lines such as Caco-2, findings subsequently confirmed in mouse intestinal organoids and human biopsy specimens [157,158]. In vivo, luminal administration of AdV vectors effectively transduced the mucosa, although transduction frequently failed to reach the deeper crypts of Lieberkühn due to physical barriers [128,130,159,160].

A major limitation of this approach lies in the high turnover rate of epithelial cells, which precludes sustained transgene expression. The episomal DNA of vectors such as AAV becomes diluted and lost during successive cell divisions. Moreover, although inflamed tissues exhibit increased susceptibility to AdV-mediated transduction in colitis models and in tissues from patients with IBD [108,157], tissue injury associated with inflammation accelerates the loss of vector genome copies, further compromising long-term therapeutic stability.

Given these constraints, the ENS constitutes a promising and more durable cellular target. Studies examining AAV-mediated transduction of ENS cells have consistently reported varying levels of myenteric neuronal transduction [161,162,163,164], often with a preferential tropism toward specific neuronal subtypes. For instance, both AAV8 and AAV9 preferentially target excitatory motor neurons and intrinsic sensory neurons, displaying weaker affinity for inhibitory motor neurons. In the SMP, the tropism of AAV8 and AAV9 is modestly biased toward VIPergic secretomotor and vasodilator neurons, and transduction efficiency tends to be lower in the colon than in the ileum [142].

EGCs, the other principal component of the ENS, also represent a compelling target. To date, only a single study has demonstrated successful EGC transduction: an intramural injection of an AAV6 vector carrying the CBh promoter into the descending colonic wall achieved transduction of approximately equal proportions (35%) of neurons and EGCs [36].

### 5.5. Choice of a Route of Administration

The various routes of administration (ROA) for adeno-associated virus (AAV) vectors have been extensively investigated. The interplay between ROA, capsid serotype, and promoter configuration has proven critical, enabling selective and efficient transduction of specific cell populations, notably ENS and intestinal epithelial cells. AAV-mediated transduction of intestinal cells has been demonstrated in mice, rats, guinea pigs, and non-human primates using multiple ROA.

#### 5.5.1. Intravenous Administration

Intravenous (IV) administration of AAV9 vectors has been performed in neonatal (2.5 × 10^9^–4 × 10^11^ vector genomes [vg] per pup) [161,165,166], juvenile (2 × 10^12^ vg per mouse) [165], and adult mice (3.3 × 10^11^ vg per mouse) [162], using Enhanced Green Fluorescent Protein (EGFP) as a reporter gene under CBh or CMV promoters. EGFP expression was detected in myenteric neurons of the ileum and colon with variable intensity, whereas no significant transduction of enteric glial cells (EGCs) was observed even with the GFAP promoter [165]. Comparable results were obtained with AAV8 and AAV6 vectors [165,166].

IV injections were also tested in neonatal guinea pigs and in *Macaca fascicularis* at postnatal days 1, 30, and 90 [167] using AAV8-EGFP-CMV vectors at doses of 2 × 10^12^ vg per animal in guinea pigs and 3–12 × 10^14^ vg per animal in primates. As observed in mice, transduction was confined to a subset of myenteric neurons, with no detectable expression in EGCs. Polyak et al. utilized the superior mesenteric artery (SMA) to administer AAV8 or AAV9-EGFP-CMV vectors (5 × 10^10^ vg per mouse), resulting in sparse transduction of presumed epithelial cells within the ileal epithelium [168]. The same group subsequently employed SMA or portal vein delivery of AAV10 vectors encoding the murine *IL10* gene under the CBA promoter in IL10^−/−^ mice (a model of enterocolitis) [168]. This approach produced robust transduction in myenteric neurons and liver cells. Overall, systemic delivery of AAV6, 8, 9, or 10 at standard gene therapy doses achieved durable transgene expression in myenteric neurons but not in EGCs.

#### 5.5.2. Enteral Administration

Enteral routes have also been explored in rodents via small bowel gavage or enema [169,170,171]. In adult mice, AAV8 or AAV9 administration by these routes resulted in negligible transduction in the ileum or colon [170]. To improve vector stability, gastric acid neutralization and protease inhibition were applied prior to peroral AAV2 administration, yielding detectable vector genomes in the stomach and small intestine of 8 out of 10 mice (versus 2 of 10 controls), although no transgene expression was observed in gastrointestinal tissue [169]. In a separate study, orogastric delivery of an AAV2-β-galactosidase vector (dose: 1.2 infectious units, vg content not reported) to lactase-deficient adult rats induced extensive transduction of epithelial and lamina propria cells [171]. Transgene expression in epithelial cells was transient due to rapid enterocyte turnover (3–5 days), but remained stable in lamina propria cells (likely immune, endothelial, or smooth muscle cells) for at least 6 months. Notably, phenotypic correction of lactase deficiency persisted for 4 months post-administration, suggesting that peroral AAV delivery can support stable protein expression.

#### 5.5.3. Intraperitoneal Administration

Unexpectedly, intraperitoneal injection of an AAV9-EGFP-CMV vector (2 × 10^11^ vg per rat) achieved transduction of colonic epithelial cells [172]. Thirty days post-injection, strong EGFP expression was observed in mucosal and submucosal cells throughout the colon, although persistence was improbable given their high proliferative turnover.

#### 5.5.4. Bowel Intramural Administration

Finally, direct intramural injection into the wall of the descending colon has been evaluated in rodents [36]. Local delivery of various AAV vectors was performed in young adult mice (2 µL, 2 × 10^9^ vg per site) and rats (5 µL, 6 × 10^9^ vg per site). Intracolonic injections of AAV5 or AAV9-EGFP-CBh in rats and AAV1, 5, or 8-EGFP-CBh in mice resulted in transduction of approximately 20–30% of local myenteric neurons, with rare EGC labeling. Increasing the viral titer from 10^12^ to 10^13^ vg/mL enhanced neuronal transduction from roughly 30% to over 70 A single injection affected approximately 47 mm^2^ of tissue, with gene expression confined to the injection site and absent from the mucosa. GFP-positive cells were not detected in the dorsal motor nucleus of the vagus or spinal cord regions; sensory neurons and dorsal root ganglia were not examined, and no vector genomes were found in the liver or spleen. Comparable transduction patterns were observed following direct AAV9 injections (5 µL, 1.2 × 10^13^ vg/mL) into the small intestine, cecum, or ascending colon of adult rats. Interestingly, intracolonic AAV6-EGFP-CBh administration achieved transduction of approximately 35% of both myenteric neurons and EGCs—currently the only reported example of efficient EGC transduction. Finally, Gore and Kitto tested 2–5 µL intracolonic injections of AAV9 vectors under various promoters in 4-week-old mice, consistently replicating these findings [36]. These injections yielded robust myenteric neuronal transduction and, importantly, AAV9-mediated transgene transfer to colon-innervating sensory dorsal root ganglia neurons, as previously documented [173,174,175,176,177].

### 5.6. The Animal and Cellular Model Challenges

The selection of an appropriate animal model remains a major challenge in preclinical gene therapy research for CD. As with other multifactorial disorders, the etiology of CD involves numerous predisposing human genetic variants in combination with yet-unidentified environmental triggers that are likely species-specific. Consequently, no single animal model faithfully reproduces the hallmark features of CD, such as transmural inflammation and the characteristic ileal predilection.

Despite these limitations, experimental pathways for gene therapy remain open. Preclinical investigations generally adopt a dual-model strategy to address complementary aspects of research: (i) studies in normal animals (rodents and non-human primates) are indispensable for assessing vector biodistribution and transgene expression patterns in healthy intestinal tissues, thereby providing preliminary data that may inform human translation; and (ii) studies in colitis models, in which inflammation is chemically induced. Chemically triggered colitis models imperfectly simulate the inflammatory component of CD. While they fail to recapitulate key CD features, they remain valuable screening systems for barrier damage and innate inflammation. For future research, the use of murine models that more closely recapitulate CD-like ileitis and transmural inflammation should be prioritized to enhance translational relevance [178]. Importantly, successful therapeutic outcomes in established colitis models remain highly informative, as they could pave the way for progress in CD-directed gene therapy, echoing the translational trajectory previously achieved with anti-inflammatory agents now in clinical use.

So-called humanized mice models may also serve as valuable platforms for testing gene therapy approaches. Indeed, the transfer of a dysbiotic microbial community from patients with Crohn’s disease (CD) has been shown to induce spontaneous inflammatory responses and CD-like ileitis or colitis in germ-free mice [179,180,181].

Beyond animal models, patient-derived epithelial organoids (PDOs) generated from colonic mucosal biopsies of multiple CD donors can retain sufficient “disease memory” to provide a relevant ex vivo system for evaluating novel therapeutic strategies [182,183,184]. However, the suitability of this model for gene therapy research remains under debate due to several significant limitations. These include the observation that organoid cultures derived from inflamed CD tissues can revert to a healthy-like phenotype [185], the absence of vascularization in organoid structures [186], the inability to model in vivo routes of vector administration, and the purely epithelial composition of current organoid systems, which excludes the enteric nervous system (ENS) and thus constrains studies on gene therapy targeting this compartment.

## 6. Prospects and Challenges for Future Gene Therapy in CD

In this section, we will focus exclusively on the use of AAV vectors, as they have become the principal platform for gene therapy [111,112]. We envision gene therapy not as a replacement but as a complement to the use of pharmacological agents that target inflammation. One potential application of gene therapy is a direct role in suppressing the intestinal inflammation, either at an early stage of the disease or during its course. This approach would rely on intestinal expression of a transgene capable of durably counteracting ongoing inflammatory processes, thereby reducing the need for surgical resection. Alternatively, driving hepatic expression of an anti-inflammatory transgene with systemic release of its product could also be beneficial in controlling intestinal disease. However, this strategy carries the risk of off-target transgene expression.

Regardless of whether expression occurs in the liver or the intestine, stringent safety standards must be met, particularly regarding regulation of circulating transgene product levels, as illustrated by the secondary effects of systemic IL-10 expression [109]. To our knowledge, studies using non-human primate models of colitis have been limited to pathophysiological investigations [187,188,189,190], and have not yet investigated intestinal any gene therapy approach. Despite the substantial financial and ethical constraints, studies in non-human primates should be prioritized, as employing a species phylogenetically closer to humans is essential for the clinical translation of gene therapy strategies.

How can future studies be designed to advance gene therapy for the benefit of CD patient? Upcoming research employing both established colitis models and emerging CD-like murine models [178] will be essential to define the optimal AAV capsid, promoter, ROA, and dosage for achieving appropriate vector biodistribution and efficient transgene expression. An intriguing question is whether a peroral route of AAV administration (a conceptual “gene pill” [171]) could be leveraged to control CD flare-ups. Even transient anti-inflammatory transgene expression, for instance within rapidly renewing epithelial cells, might confer therapeutic benefit under such conditions.

AAV-based gene therapy may also have valuable applications for the large subset of CD patients who require ileocolic resection and remain at high risk of postoperative recurrence. Building on the pioneering preclinical findings of Benskey et al. [36], a promising surgical strategy would consist of injecting the vector directly into the intestinal wall of non-resected segments adjacent to the anastomosis to prevent subsequent inflammatory relapse. Given that postoperative recurrence typically arises at the ileocolic anastomosis and neo-terminal ileum, the size of the target region is compatible with near-complete coverage through multiple localized vector injections. In the study by Benskey et al. [36], the transgene expression area extended approximately 50 mm^2^ around each injection site, with robust expression observed in both enteric neurons and glial cells. Notably, achieving full surface coverage near the anastomosis may not be necessary, as the transgene product—such as a cytokine—can diffuse beyond the immediate area of expression, potentially providing broader local transgene expression.

## 7. Conclusions

Despite the complexity of the genetic and inflammatory mechanisms underlying CD, which precludes straightforward single-gene replacement strategies, this review underscores the considerable therapeutic potential of gene therapy. The targeted delivery of genes encoding anti-inflammatory chemokines or cytokines into enteric neurons and/or glial cells (EGCs) using AAV vectors represents a promising approach to modulate chronic intestinal inflammation. Beyond the treatment of CD itself, gene therapy could have an immediate and substantial clinical application in the prevention of postoperative recurrence, a persistent and challenging complication. In this context, the experimental strategies discussed naturally extend to models of colitis and other IBD. In conclusion, by enabling localized and sustained modulation of the intestinal immune response, gene therapy offers a forward-looking and mechanistically grounded avenue for improving the management not only of CD but also of other IBDs and their associated complications. More generally, it has become clear that gene therapy progress will require not only advances in biotechnology, but immunology, pharmaceutical development, well-designed and ethical clinical trials demonstrating significant clinical benefits and long-term safety. We are conscious in this respect that Rome was not built in a day.

## Abbreviations

The following abbreviations are used in this manuscript:

AAV	Adeno-associated viral vectors
AdV	Adenovirus
CAG	CMV early enhancer/chicken β-actin/short β-globin
CBA	Chicken β-actin
CBh	CBA hybrid
CD	Crohn’s disease
CMV	Cytomegalovirus
Cnp	2′,3′-cyclic-nucleotide 3′-phosphodiesterase
CNS	Central nervous system
CRC	Colorectal cancer
CSF	Colony-stimulating factor
eGAPS	enteric glia associated with psychological stress
EGF	Epidermal growth factor
EGFP	Enhanced green fluorescent protein
EGCs	Enteric glial cells
ENS	Enteric nervous system
GDNF	Glial cell line-derived neurotrophic factor
GFAP	Glial fibrillary protein
GWAS	Genome-wide association studies
IBD	Inflammatory bowel diseases
IDLV	Integrase-defective lentiviral vectors
IFN	Interferon
IL	Interleukin
ILC	Innate lymphoid cells
IV	Intravenous
LV	Lentivirus
MAG	Myelin-associated glycoprotein
Mbp	Myelin basic protein gene
MLV	Murine leukemia virus
MP	Myenteric plexus
Mpz	Myelin-specific myelin protein zero
PGK	Phosphoglycerate kinase
Plp	Proteolipid protein
PLP-1	Proteolipid protein 1
PNS	Peripheral nervous system
sCAG	Short CMV early enhancer/chicken β-actin/short β-globin
SIN	Self-inactivating
SMA	Superior mesenteric artery
SMP	Submucosal plexus
SOX10	SRY-Box Transcription Factor 10
SYN	Synapsin
TGF	Transforming growth factor
TNBS	2,4,6-trinitrobenzene sulphonic acid
TNF	Tumor necrosis factor
VIP	Vasoactive intestinal peptide
VSV-G	Vesicular stomatitis virus G protein
